# Factors associated with divorce from first union among women in Ethiopia: Further analysis of the 2016 Ethiopia demographic and health survey data

**DOI:** 10.1371/journal.pone.0244014

**Published:** 2020-12-15

**Authors:** Gizachew Worku Dagnew, Melash Belachew Asresie, Gedefaw Abeje Fekadu, Yared Mulu Gelaw

**Affiliations:** 1 Department of Reproductive Health and Population Studies, School of Public Health, College of Medicine and Health Science, Bahir Dar University, Bahir Dar, Ethiopia; 2 Department of Health Services Management, School of Public Health, College of Medicine and Health Sciences, Bahir Dar University, Bahir Dar, Ethiopia; University of Cape Coast, GHANA

## Abstract

**Background:**

Globally, divorce is a common phenomenon in couples' marital life. As a result, many divorced couples and their children face several social, economic, and health problems after dissolution. There is little information on the magnitude and determinants of divorce in developing countries including Ethiopia. Therefore, this study aimed to estimate the prevalence of divorce from the first union and its predictors among reproductive-age women in Ethiopia.

**Methods:**

We used the 2016 Ethiopia demographic and health survey data for this analysis. The survey was a community-based cross-sectional study conducted from January 18 to June 27, 2016. The survey employed a two-stage stratified cluster sampling technique. A total of 11,646 ever-married women were included in the analysis. Bivariate and multivariable logistics regression was done to identify the determinants of divorce from the first marriage. A p-value < 0.05 was used to declare statistical significance.

**Results:**

About 25% (95%CI: 23.4% - 26.6%) ever-married women were divorced from their first marital relationship. Women who were married at age < 15 years (AOR = 1.34; 95%CI: 1.07–1.68), urban women (AOR = 1.69; 95%CI: 1.22–2.35), women who did not attend formal education (AOR = 4.36; 95%CI: 3.14–6.05), women who were employed (AOR = 1.51; 95%CI: 1.31–1.73), and being childless (AOR = 1.34; 95%CI: 1.07–1.69) had higher odds of experiencing a divorce. Similarly, women who experienced partner violence, women with no house ownership, and women in the Amhara region had higher odds of divorce from their first marital union. Conversely, women in Oromia, SNNPR, the metropolis, and the pastoral regions had lower odds of divorce from their first marital union.

**Conclusion:**

Divorce from the first marriage is high in Ethiopia. Preventing early marriage and partner violence and promoting girls’ education would reduce the divorce rate in Ethiopia.

## Introduction

Marriage is a phenomenon that most people around the world pass through [1, 2]. It is considered one of the most important forms of social support for adults [3]. Population-based studies have found that most adults will marry at some point in their lifetime [2]. There were around 4.8 crude marriage rates per 1,000 people in the organization for economic co-operation and development (OECD) member countries [4]. Marriage is more than being together, rather a man and woman together as husband and wife to be father and mother to any children that their union produces. It is society's minimum restrictive means of safeguarding the welfare of children [2, 5]. However, marriage may not continue until the end of the couple's life. Most of the marital unions ended with the separation of a husband and wife (divorce). Divorce is a final legal dissolution of a marriage; consult on the parties the right to remarriage under civil, religious, and/or other provisions, according to the laws of each country [5, 6].

In the recent decade, the rate of divorce increased globally [7]. A study from 33 Sub-Saharan Africa (SSA) countries indicated that on average one-fourth (25%) of the first union ended with divorce, which ranges from 6.9% in Mali to 47.1% in Congo (Brazzaville) [8]. Some small-scale studies in Ethiopia also showed that more than one-third of women were divorced from their first union [9, 10]. According to literature; age at marriage, employment status, partner abuse, globalization, sexual satisfaction, and economic problems were the predictors for divorce [8, 11–15].

Divorce is taken as a solution for abusive and violent marital life [16]. However, it has several socio-economic and health problems for the divorced couple and their children [7, 16–19]. Literature from developed countries confirmed that children with divorced parents experienced more mental and physical health problems than do children with married parents [20–22]. The consequences of divorce among children of developing regions are even more severe than in the high-income regions [23]. After divorce children belonging to lower socio-economic groups experience greater hardships than the high socio-economic groups. Do these hardships; they face adjustment problems. This decline in socio-economic status is directly linked to a variety of problems experienced by the child, such as psychological maladjustment and behavioral difficulties in school [24].

A multinational study conducted in SSA countries showed an increased risk of death (ranging from 36% in the Democratic Republic of the Congo to 75% in Kenya) of children from divorced parents. In Ethiopia and Malawi, children whose mothers were in a new union were at a high risk of dying relative to children whose mothers were continuously married [25]. A study from different African countries showed that having divorced parents was associated with a higher risk of under-five mortalities. The remaining surviving children were more likely to be stunted, start school late, and have poor educational attainment compared to children from married parents [26–29].

In the rural parts of Ethiopia, children from divorced parents have poor school performance [30]. Another finding from the Oromia region indicated that divorced women were more likely to experience high levels of community stigma [31]. As a response to community stigma, women may marginalize themselves from the community and face various economic, social, and health problems.

Divorce is the right and decisions of the two couples in Ethiopia. However, it may have multiple unwanted effects for the divorced couple, the children, the family, and the community at large. In Ethiopia, there are no national-based studies as well as strategies about divorce, though there are few urban-based small-scale studies. Therefore, an investigation of factors associated with divorce using national data is paramount important. Hence, this analysis was done to estimate the magnitude of divorce and its predictors among reproductive-age women in Ethiopia. The findings of this study can help to design a preventive strategy for divorce.

## Material and methods

### Data source, study design, and setting

In reporting this study, we adopted the Strengthening the Reporting of Observational Studies in Epidemiology (STROBE) checklist for cross-sectional studies.

The study used the 2016 Ethiopia demographic and health survey (EDHS) data, collected from January 18 to July 27, 2016, from all administrative regions. This survey is the fourth DHS conducted in Ethiopia. It was a community-based cross-sectional survey. A total of 15,683 reproductive age (15–49 years) women were included in the survey. The survey covered all administrative regions. Ethiopia has nine geographical regions and two administrative cities [32].

### Study population and sampling techniques

The 2016 EDHS used a two-stage stratified cluster random sampling technique to make sure the representativeness of the sample by regions and residence [32]. Initially, each region was stratified into urban and rural areas yielding 21 sampling strata. After stratification, a total of 645 enumeration areas (202 in urban areas and 443 in rural areas) were selected with probability proportional to the enumeration area size. A household listing operation was done from September to December 2015. Then, 28 households from each cluster were selected using a systematic random sampling technique [32].

For this study, we used the women data set. All ever-married women were the participants for this study; never-married women were excluded from the analysis. Based on these criteria, a total of 11,646 women were included in the final model [32].

### Study variable and measurements

#### Outcome variable

Divorce from the first marriage is the outcome of this analysis. A variable has two outcomes (yes/no). It was measured based on the woman’s self-report of divorce during the survey or have a history of more than one union formation (re-marriage history).

#### Explanatory variables

The socio-demographic variables; include women’s age at first marriage (< 15, 15–18 and ≥ 19 years), residence (urban and rural), religion (Christian, Muslim, and others), women’s education status (no formal education, primary, and secondary or above), employment status (employed and not employed), having children (No/Yes), giving birth before marriage (No/Yes), region (Tigray, Amhara, Oromia, South Nation Nationality People republic (SNNPR), living in the pastoral region and the three metropolis regions, wealth index (poor, middle and rich), land ownership (No/Yes), housing ownership (No/Yes), experienced partner violence (No/Yes), and marital control behavior by husband (No/Yes).

The metropolis regions include the three urban-based administrative regions (Harari, Addis Ababa, and Dire Da’wa). The pastoral regions include four pastoral developing regions (Afar, Somali, Benishangul, and Gambella). Population stability of living and human development index was used to merge these regions. The regions categorized as metropolis have a high human development index compared to other regions, on the other hand, the pastoral regions are found in the lowland areas of Ethiopia, mostly move from place to place with their livestock in search of grass and water. The rest unmerged regions are found in the highland area of the country; farming is the main occupation of those most settled populations.

Experience of partner violence; if women reported any of the specified acts of physical, sexual, or emotional violence committed by their husband/partner [32].

Marital control behavior; women had marital control behavior when the women reported that her husband/partner demonstrated at least one of the following controlling behaviors: is jealous or angry if she talks to other men, frequently accuse her of being unfaithful, not permit her to meet her female friends, tried to limit her contact with her family, and insisted on knowing where she is at all times [32].

### Statistical analysis

We used STATA 14.0 for this analysis. By using descriptive statistics, summary measures of the median with interquartile-range, frequency, percentages, and chi-square statistics were computed and presented via a table and text. Data were weighted using women’s data weighting variable (V005/1,000,000) as recommended by the EDHS to ensure the representativeness of the survey by regions and residence and to account for non-response. Furthermore, the analysis was adjusted to account for the complex survey design and robust standard errors (stratification and clustering) using the ‘svy’ command in Stata.

Bivariate logistics regression analysis was conducted to select the candidate variables for multivariable logistics regression. Variables with a p-value ≤ 0.2 in the binary logistic regression analysis were included in the multivariable logistic regression model. Multicollinearity between each explanatory variable was checked by using the ‘pwcorr’ command in Stata. Model fitness was checked by using the Hosmer-Lemeshow goodness of fit test (p>0.05) [33].

The multivariable analysis was done to identify factors associated with divorce. Only variables with a p-value of less than 0.05 in the multivariable analysis were considered statistically significant. The adjusted odds ratios (AOR) with corresponding 95% confidence interval were presented in the results section.

### Ethical approval

The 2016 EDHS protocol was reviewed and approved by the National Ethics Review Committee of the Federal Democratic Republic of Ethiopia, Ministry of Science and Technology, and the Institutional Review Board of ICF International. The STATA format data was downloaded from the DHS program with permission.

## Results

### Socio-demographic characteristics of women

A total of 11,646 ever-married reproductive-age (15–49 years) women were included in the analysis. The median age at marriage (±interquartile range (IQR)) of the study participants was 16.0 (±3) years with a minimum of 10 years and a maximum of 43 years. About 81.9% of women were rural residents. Nearly 61% of women did not attend formal education. Around 90% of women were from major regions (Oromia 38.1%, Amhara 24.8%, SNNPR 19.8%, and Tigray accounts 7.3%). More than 14% of women reported that they experienced some forms of partner violence (emotional, physical, or sexual). About 19% of ever-married women reported that they experienced at least one type of marital control behavior (Table 1).

**Table 1:** Sociodemographic characteristics of ever-married women in Ethiopia, 2016 EDHS.

Variables (n = 11,646)	Categories	Frequency	Percentage (%)
Age in years at first marriage/union	<15	3,055	26.2
	15–18	5,404	46.4
	≥19	3,187	27.4
Residence	Urban	2,102	18.1
	Rural	9,544	81.9
Region	Tigray	847	7.3
	Amhara	2888	24.8
	Oromia	4433	38.1
	SNNPR	2310	19.8
	The three metropolises	543	4.7
	Pastoralist region	626	5.4
Wealth index	Poor	4,504	38.7
	Middle	2,324	20.0
	Rich	4,819	41.4
Educational status	No formal education	7,059	60.6
	Primary	3,351	28.8
	Secondary or above	1,237	10.6
Religion	Christian	7,552	64.8
	Muslim	3,907	33.5
	Others	188	1.6
Women employment	Not employed	5,679	48.8
	Employed	5,968	51.2
Women with children	No	1,153	9.9
	Yes	10,494	90.1
Women give birth before marriage	No	10,941	93.9
	Yes	705	6.1
A history of abortion	No	10,429	89.5
	Yes	1,217	10.5
Had marital control	No	9,9426	80.9
	Yes	2,220	19.1
History of partner violence	No	9,907	85.1
	Yes	1,740	14.9
Land ownership	No	5,556	47.7
	Yes	6090	52.3
House-ownership	No	4,187	36.0
	Yes	7459	64.0

### Divorce from marriage

The analysis identified that 24.9% (95%CI: 23.4% - 26.6%) of ever-married women had experienced a divorce. Out of women who experience divorce, 34.2% of them were divorced at the time of the survey. About 77% of them also reported that they divorced more than once.

### Factors associated with divorce

The multivariable logistics regression analysis showed that age at marriage, residence, region, educational status, employment status, history of abortion, history of partner violence, and house-ownership had a statistically significant association with divorce. Women who were married at age < 15 years had higher odds (AOR = 1.34; 95%CI: 1.07–1.68) of divorce compared to those who were married at age ≥ 19 years. Urban women had higher odds (AOR = 1.69; 95%CI: 1.22–2.35) of divorce from marital union compared to rural women. Women who did not attend formal education (AOR = 4.36; 95%CI:3.14–6.05) and those who attended primary education (AOR = 3.17; 95%CI: 2.29–4.38) had high odds of divorce compared to those who attended secondary or higher level of education. Women who were living in the Amhara region had high odds of divorce compared to women who were living in the Tigray region (AOR = 1.50; 95%CI: 1.18–1.89). On the other hand, women who were living in Oromia (AOR = 0.31; 95%CI: 0.23–0.41), SNNPR (AOR = 0.27; 95%CI: 0.21–0.36), the metropolis (AOR = 0.58; 95%CI: 0.42–0.79), and the pastoral regions (AOR = 0.36; 95%CI: 0.27–0.48) had lower odds of divorce compared to those who were living in Tigray region. Women who were employed had high odds of divorce compared to women who were not employed (AOR = 1.51; 95%CI: 1.31–1.73). Women with no children had higher odds of divorce compared to women with children (AOR = 1.34; 95%CI: 1.07–1.69). Women who reported a history of some forms of partner violence had higher odds of divorce compared to their counterparts (AOR = 1.60; 95%CI: 1.30–1.98). Women who were living in rented houses (AOR = 1.81; 95%CI: 1.50–2.19) had higher odds of divorce compared to those who were living in their own houses (Table 2).

**Table 2:** Factor associated with divorce among ever-married women in Ethiopia, 2016 EDHS.

Variables (n = 11,646)	Divorced/separated			
	Yes (%)	No (%)	COR (95%CI)	AOR (95%CI)
**Age at first marriage (in years)**				
<15	33.0	67.0	1.84(1.51–2.23) ***	1.34(1.07–1.68) **
15–18	22.7	77.3	1.10(0.92–1.30)	0.96(0.80–1.16)
≥19	21.1	78.9	1	1
**Place of residence**				
Rural	29.0	71.0	1	1
Urban	24.0	76.0	1.29(1.04–1.60) *	1.69(1.22–2.3) **
**Educational status**				
No formal education	27.5	72.5	2.20(1.73–2.80) ***	4.36(3.14–6.05) ***
Primary	23.3	76.7	1.77(1.35–2.31) ***	3.17(2.29–4.38) ***
Secondary or above	14.7	85.3	1	1
**Wealth index**				
Poor	24.3	75.7	0.99(0.82–1.19)	1.10(0.89–1.36)
Middle	26.8	73.2	1.12(0.91–1.38)	1.18(0.94–1.48)
Reach	24.6	75.4	1	1
**Region**				
Tigray	38.5	61.5	1	1
Amhara	47.1	52.9	1.42(1.14–1.78) **	1.50(1.18–1.89) ***
Oromia	15.3	84.7	0.29(0.23–0.37) ***	0.31(0.23–0.41) ***
SNNPR	12.8	87.2	0.23(0.18–0.31) ***	0.27(0.21–0.36) ***
Metropolises	27.0	73.0	0.59(0.47–0.75) ***	0.58(0.42–0.79) ***
Pastoralist regions	15.8	84.2	0.30(0.24–0.38) ***	0.36(0.27–0.48) ***
**Religion**				
Christian	28.5	71.5	1	1
Muslim	18.8	81.2	0.58(0.46–0.73) ***	0.90(0.72–1.13)
Others	7.8	92.2	0.21(0.06–0.70) *	0.42(0.12–1.47)
**Women employment**				
Not employed	19.1	80.9	1	1
Employed	30.5	69.5	1.86(1.60–2.15) ***	1.51(1.31–1.73) ***
**Women with children**				
No	33.0	67.0	1.56(1.27–1.90) ***	1.34(1.07–1.69) *
Yes	24.0	76.0	1	1
**A history of abortion**				
No	24.2	75.8	1	1
Yes	30.8	69.2	1.39(1.15–1.69) **	1.27(1.03–1.58) *
**Had marital control behavior**				
No	24.4	75.6	1	1
Yes	27.1	72.9	1.15(0.98–1.35)	0.96(0.78–1.18)
**History of partner violence**				
No	23.6	76.4	1	1
Yes	32.5	67.5	1.62(1.36–1.92) ***	1.60(1.30–1.98) ***
**Land ownership**				
No	25.5	74.5	1,06(0.91–1.24)	0.95(0.79–1.16)
Yes	21.6	78.4	1	1
**House ownership**				
No	28.5	71.5	1.34(1.15–1.55) ***	1.81(1.50–2.19) ***
Yes	21.4	78.6	1	1

*** p<0.001,

** p<0.01,

* p<0.05, COR: Crude Odd Ratio, CI: Confidence Interval, AOR: Adjusted Odd Ratio.

## Discussion

This study examined the prevalence of divorce from a first marriage and associated factors among ever-married women in Ethiopia. About 25% of ever-married women were experienced divorce from their first marriage. In a country like Ethiopia that gives high value to marriage and has different social norms that are used to solve disagreements between couples, this finding is unexpectedly high, though some countries have consistently high or more divorce rates. This study finding was consistent with a multinational study finding done among 33 SSA countries [8]. However, it is lower than the study from Gondar (36.8%) and Bahir Dar cities Ethiopia (46.5%) [9, 10]. The reason for the`lower prevalence of divorce in this study compared to other studies might be the difference in place of residence; rural women have a lower rate of experiencing divorce compared to urban. Around 79% of women were rural residents in the current study while it is below half in the aforementioned studies [9, 10]. The other plausible reason for the lower prevalence of divorce in the current study might also be a difference in women's employment status; employed women have a higher prevalence of divorce [8, 34].

The study revealed that women's age at first marriage/union was statistically associated with divorce. The odds of experiencing divorce was higher among women whose age at first marriage was less than 15 years compared to those whose age at first marriage was 19 years or more. This finding was in line with other studies [9, 12, 35]. The reason for this might be the difference in maturity level and preparation for marriage. Women married before the age of 15 years have low knowledge and plan for their marriage as compared to those who got married at age 19 years or more [36, 37]. And they are more likely exposed to physical and psychological trauma [38, 39]. Early married girls also face sexual dysfunction in their later life because of a higher rate of experiencing forced first marital sex than those married at a late age [37]. Due to immaturity and loss of interest, those girls fail to plan and manage their families. Hence, they are more likely to run away from marriages [13, 39, 40].

The study showed that urban women had higher odds of divorce compared to their rural counterparts. This finding in line with other studies [8, 41, 42]. The reason for this might be the difference in housing ownership and cost conflict between urban and rural couples. This is higher among urban women compared to rural counterparts [43]. The other reason might also be the divorced women in rural areas are more mistreated than urban counterparts, and they refrain from divorce [44]. On the other hand, globalization situates its effects on urban women than rural. With increased prominence on personal preference and rational choice, dramatic changes of a marital union may occur within a limited time. Economic affluence and personal autonomy have been accompanied by increased divorce and marital infidelity [45].

This analysis showed that women who were living in the Amhara region had higher odds of divorce compared to the reference region (Tigray). On the other hand, those who were living in other regions had lower odds of divorce compared to women from the Tigray region. A possible explanation for this might be age difference at first marriage. The smallest mean-age at first marriage was reported from the Amhara region (15.9 years) followed by Tigray (16.6 years) and the maximum mean-age at first marriage was reported from the Metropolis region (20.1 years) followed by SNNPR (17.4 years). Age at first sexual intercourse might contribute to the regional difference of divorce. Women who began having sex as teens are more likely to divorce: mainly if the first sex is unwanted [46]. The other plausible explanation might also be regional differences in women's education. Women from most city sett-up regions have a higher level of education; this might extend the age of marriage.

Women who did not attend formal education or attended primary education had higher odds of divorce compared to women who attended secondary or higher level of education. This finding was consistent with other studies [8, 47, 48]. This is explained as girls' education can increase women's age at marriage; increase the age of marriage would reduce divorce. Different global and national strategies focused on girls' education to prevent early-marriage [49–51]. According to this study, the mean age at marriage was relatively higher among women who attended secondary or higher education (20 years) compared to those who did not attend formal education (16.4 years). Women whose age exceeding 18 years has higher knowledge and plan for marriage than age below 18 years [36]. Educated women are also less influenced by external pressure to decide their first marriage. These might contribute to a higher rate of marital stability and less divorce rate among women who attend secondary or above education compared to no or less educated women.

The other finding of this study was that employed women had higher odds of divorce compared to unemployed women. This was supported by other studies [8, 34]. For unemployed women, divorce could lead to high economic hardships than employed women. Women's employment increases economic opportunity for disruption of unhappy marriage [52]. A study in Israel showed that when the wife earned equal or more than the husband, divorce was more likely [53]. On the other hand, unemployed women are economically dependent on their husbands' income, and they fear divorce due to their economic insecurity. As a result, many unemployed women might be living with a problem in their marital life compared to their counterparts.

Women who did not have a child had higher odds of divorce. The other finding of this study also women who had a history of abortion had higher odds of divorce compared to their counterparts. In most African countries including Ethiopia, marriage is measured by the number of children they bear. In most cultures, if marriage is not blessed by children, the marriage is considered not achieved its aim and cause for divorce [54, 55]. If the couple's interest in childbearing is varied, or unable to conceive offspring due to abortion or other reasons may precipitate to end a marriage [56, 57]. Infertility is one of the consequences of abortion.

This study showed that women who experienced some forms of partner violence had higher odds of divorce compared to their counterparts. This finding was supported by studies done in Gondar city Ethiopia and Uganda [9, 58]. Divorce is a final strategy of coping with spousal violence if other strategies do not work; like correcting causes of violence, or living with the problem [59].

One of the strengths of this study is the use of nationally representative data with a large number of participants. This might have a better prediction of parameters. The other strength also this study is the first effort at understanding the level of divorce and its predictors at the national level. However, this study has few limitations: this study used secondary data; the design of the national DHS does not allow to establish the temporal order of divorce and its correlates. Particularly for the indicators of employment status, wealth index, and residence. There is a possibility that the values on these factors may have changed in response to divorce. This analysis also did not include important variables that can explain the rate of divorce. This includes; marital and sexual satisfaction, duration of the marriage, and third-person interference in the couples' marital life.

## Conclusions

The proportion of Ethiopian women who experienced divorce was high. Increasing girl's education, preventing child marriage (age < 15 years), abortion, and partner violence against women would reduce the rate of divorce in the country. Living in urban areas and being childless increased the prevalence of divorce in Ethiopia.

## Supporting information

S1 File (DOCX)

## Abbreviations

AOR: Adjusted Odds Ratio
CI: Confidence Interval
EDHS: Ethiopian Demographic and Health Survey
