# Supplementary material for: Factors associated with divorce from first union among women in Ethiopia: Further analysis of the 2016 Ethiopia demographic and health survey data
Source: PLoS One. 2020 Dec 15;15(12):e0244014. doi: 10.1371/journal.pone.0244014 (PMC7737979; doi:10.1371/journal.pone.0244014)
Supplement: S1 File — (DOCX) [file pone.0244014.s001.docx]

## **Materials and methods**

## Explanatory variable coding and measurement

Table 1: List of variables used for analysis and their definition and measurement based on the EDHS 2016 report

| Variable | Survey question and category | Coding and measurement |
| --- | --- | --- |
| Age at marriage/union | How old were you when you first started living with a man? | The age of women was collected as continuous data in the DHS and recoded as; < 15 years, 15-18 years, ad ≥ 19 years for this study. |
| Residence | Place of residence (urban or rural) | Use the same code and category |
| Region | The region was coded as; Tigray, Afar, Amhara, Oromia, Somali, Benishangul-Gumuz, SNNPR, Gambela, Harari, Addis Ababa, and Dire Dawa. | The region category recoded as Tigray, Amhara, Oromia, SNNPR, Pastoral regions (Somali, Afar Benishangul-Gumuz, and Gambela), and metropolis (Harari, Addis Ababa, and Dire Dawa). |
| Wealth index | Have five categories; poorest, poorer, middle, rich, ad richest | It is recorded as poor (poorest and poor), middle, and rich(rich and richest) |
| Educational status | The highest education level attended.  It has four categories: No education, Primary, Secondary, and Higher. | Education recorded three categories; No formal education (No education), primary education, and secondary or higher education (secondary and higher) |
| Religion | Religio has six categories; orthodox, Catholic, protestant, Muslim, traditional, and others | Which is measured in three categories; Christian (orthodox, catholic, protestant), Muslim ad others (traditional and others) |
| Women employment | Respondents occupation was categorized as; not working, professional/technical/managerial, clerical, sales, agricultural – employee, services, skilled manual, unskilled manual, and others | Recoded as in two categories; no working recorded as unemployed and the rest was coded as employed. |
| Women with children | The total number of children ever born. | Recoded as No/Yes; ‘No’ when the respondent reports zero children, and ‘Yes’ for respondents reported one or more children |
| Women give birth before marriage | The interval between the first marriage and first birth in months. If the first birth was before the first marriage then this variable is coded 996 "Negative interval | Recoded as No/Yes; ‘No’ for respondents reported no birth or positive birth interval, ‘Yes’ if respondents reported ‘Negative birth interval.’ |
| A history of abortion | Ever had a terminated pregnancy? No/Yes | Use the same code and category |
| Had marital control | Number of women who say “yes” when asked about each of the six different types of the specified marital control behaviors displayed by their husbands/partners   1. jealous or angry if she talks to other men; 2. frequently accuses her of being unfaithful; 3. does not permit her to meet her female friends; 4. tries to limit her contact with her family; 5. insists on knowing where she is at all times; 6. does not trust her with money. | This variable is recorded as No/Yes; ‘Yes’ if the woman says “Yes” at least for one or more different types of marital control behavior, otherwise recoded as ‘No.’ |
| History of partner violence | Assess in three sub-themes with 16 survey questions. Each survey question has 4 categories;0. never, 1. often, 2. sometimes, and 3, yes, but not in the last 12 months   1. Physical violence; It was assessed by the following 08 survey questions  - Spouse ever pushed, shook, or threw something, - Spouse ever slapped. - Spouse ever punched with a fist or something harmful. - Spouse ever kicked or dragged. - Spouse ever tried to strangle or burn. - Spouse ever threatened with a knife/gun or other weapons. - Spouse ever attacked with a knife/gun or other weapons. - Spouse ever twisted her arm or pulled her hair  1. Emotional violence; It has 3 survey questions.  - Spouse ever humiliated her. - Spouse ever threatened her with harm. - Spouse ever insulted her or made her feel bad  1. Sexual violence;  - Spouse ever physically forced sex when not wanted. - Spouse ever forced other sexual acts when not wanted - ever been physically forced to perform sexual acts respondent didn't want to - The previous husband ever hit, slap, kick or physically hurt the respondent - The previous husband physically forced to have sex or to perform sexual acts | Experienced partner violence was coded as No/Yes; “Yess” if women reported any of the specified acts of physical, sexual, or emotional violence committed by their husband/partner, if not any recoded as “No” |
| Land ownership | Owns land alone or jointly; it has four categories.  Does not own, alone only, jointly only, and both alone and jointly | It was recorded as No/Yes; “Yes” if the women reported one of the following; alone only, jointly only, or both alone and jointly |
| House ownership | Own a house alone or jointly; it has four categories.  Does not own, alone only, jointly only, and both alone and jointly | It was recorded as No/Yes; “Yes” if the women reported one of the following; alone only, jointly only, or both alone and jointly |

# Results

## Mean age at marriage by background characteristics

The mean age of women at marriage/union was relatively lower among women's age category 15-24 years compared to other age categories. Mean-age at marriage was also lower among rural women compared to urban women. Similarly, the mean age at marriage was varied by the women’s living region. And the highest mean-age at marriage was observed among women who lived in the metropolis region followed by SNNPR, and the lowest mean-age was observed among women who lived in the Amhara region followed by Tigray. Women no having formal education have a relatively lower mean-age at marriage compared to those who have primary or above education. However, mean-age at marriage had no comparative disparity between women’s religion and employment status (Table2).

Table2: Mean-age at marriage by women’s background characteristics of ever- married/ in-union women in Ethiopia, 2016 EDHS.

| Variables (n=11,646) | Categories | Mean age at marriage (95%CI) |
| --- | --- | --- |
| Current Age in years | 15-24 | 16.4(16.3-16.5) |
|  | 25-34 | 17.2(17.1-17.3) |
|  | ≥35 | 17.2(17.0-17.3) |
| Residence | Urban | 18.5(18.2-18.8) |
|  | Rural | 16.7(16.6-16.8) |
| Region | Tigray | 16.6(16.4-16.8) |
|  | Amhara | 15.9(15.7-16.2) |
|  | Oromia | 17.2(17.0-17.4) |
|  | SNNPR | 17.4(17.2-17.6) |
|  | The three metropolises | 20.1(19.9-20.3) |
|  | Pastoralist region | 17.1(17.0-17.2) |
| Wealth index | Poor | 16.6(16.5-16.7) |
|  | Middle | 16.6(16.5-16.8) |
|  | Rich | 17.6(17.5-17.7) |
| Educational status | No formal education | 16.4(16.3-16.5) |
|  | Primary | 17.1(17.0-17.2) |
|  | Secondary or above | 20.0(19.8-20.2) |
| Religion | Christian | 17.1(17.0-17.2) |
|  | Muslim | 16.9(16.8-17.0) |
|  | Others | 16.8(16.3-17.2) |
| Women employment | Not employed | 17.0(16.9-17.1) |
|  | Employed | 17.0(16.9-17.1) |
| Experienced partner violence | No | 17.0(16.9-17.1) |
|  | Yes | 16.8(16.6-17.0) |
